# Aspherical, Nano-Structured Drug Delivery System with Tunable Release and Clearance for Pulmonary Applications

**DOI:** 10.3390/pharmaceutics16020232

**Published:** 2024-02-05

**Authors:** Tomas Pioch, Thorben Fischer, Marc Schneider

**Affiliations:** Department of Pharmacy, Biopharmaceutics and Pharmaceutical Technology, Saarland University, 66123 Saarbrücken, Germany; tomas.pioch@uni-saarland.de (T.P.); thorben_fischer@gmx.net (T.F.)

**Keywords:** microrods, pulmonary drug delivery, cell interactions, particle shape, disintegration

## Abstract

Addressing the challenge of efficient drug delivery to the lungs, a nano-structured, microparticulate carrier system with defined and customizable dimensions has been developed. Utilizing a template-assisted approach and capillary forces, particles were rapidly loaded and stabilized. The system employs a biocompatible alginate gel as a stabilizing matrix, facilitating the breakdown of the carrier in body fluids with the subsequent release of its nano-load, while also mitigating long-term accumulation in the lung. Different gel strengths and stabilizing steps were applied, allowing us to tune the release kinetics, as evaluated by a quantitative method based on a flow-imaging system. The micro-cylinders demonstrated superior aerodynamic properties in Next Generation Impactor (NGI) experiments, such as a smaller median aerodynamic diameter (MMAD), while yielding a higher fine particle fraction (FPF) than spherical particles similar in critical dimensions. They exhibited negligible toxicity to a differentiated macrophage cell line (dTHP-1) for up to 24 h of incubation. The kinetics of the cellular uptake by dTHP-1 cells was assessed via fluorescence microscopy, revealing an uptake-rate dependence on the aspect ratio (AR = *l*/*d*); cylinders with high AR were phagocytosed more slowly than shorter rods and comparable spherical particles. This indicates that this novel drug delivery system can modulate macrophage uptake and clearance by adjusting its geometric parameters while maintaining optimal aerodynamic properties and featuring a biodegradable stabilizing matrix.

## 1. Introduction

The subject of pulmonary drug delivery has been the focus of intense research, especially in the context of chronic respiratory diseases (CRD) such as asthma, COPD (chronic obstructive pulmonary disease), and lung cancer. Accounting for over 50 deaths per 100,000, CRDs are the third-highest cause of death worldwide, with a continuously rising incidence [1]. The recent COVID-19 pandemic has further underlined the importance of new developments, introducing unpredictable new challenges alongside existing ones [2].

Initially, the drug must reach its target within the lungs, a process influenced mainly by particle size and shape [3]. Subsequently, the active ingredient must be liberated in the small amount of fluid available before being cleared by mucociliary action in the upper airways and macrophage clearance in the lower airways [4].

These obstacles are only enhanced by CRDs: A chronic inflammation state, induced by released cytokines from macrophages, leads to bronchial obstruction and fibrosis [5]. This results in irregular deposition patterns, making the drug delivery less effective and more unpredictable [6]. Furthermore, upregulated phagocytic activity decreases the available drug amount [7,8]. This creates the need for an efficient and targeted drug delivery system, delivering an effective dose directly to its target. Therefore, advantage is taken of the benefits of pulmonary drug administration such as a large surface area, thin and highly permeable membranes, a high blood flow for fast absorption, as well as relatively low enzymatic activity, and no first-pass effect for a high bioavailability of the drug [9,10]. Pulmonary drug delivery via inhalation devices also avoids the disadvantages of invasive drug administration such as discomfort and high costs, thus increasing cost-effectiveness and patient compliance [11]. Using this administration route, the pharmaceutic treatment of the two most common CRDs, COPD and asthma [12], typically includes SABAs (short-acting beta-2-agonists) for short-term relief and LABAs (long-acting beta-2-agonists or LAMAs (long-acting muscarinic antagonist, COPD only), often supplemented with an ICS (inhaled corticosteroid) for more severe cases [13,14].

The utilization of nanoparticles offers a solution to some of these challenges, with their mucus-penetrating capabilities and rapid and controlled drug release [15]. However, their small size poses challenges for optimal drug delivery in dry powder form like hampered flowability [16], and due to diffusion issues after aerosolization [17]. Nebulizer formulations, while more effective in this regard, introduce stability issues, present potential contamination risks, and can lower patient compliance [18]. By incorporating nano-sized particles into a microcarrier [19], it is possible to take advantage of their benefits while solving the aerodynamic challenges. Additionally, every part of the carrier system can be optimized for drug delivery: the flexible packing of the submicron structure with API-loadable gelatine nanoparticles or mesoporous SNPs [20,21] and the capability to use the stabilizing surface for gene delivery like siRNA and pDNA [22,23] have already been shown.

Another emerging area of interest is the modification of the drug delivery system’s shape [21,24,25] drawing inspiration from nature, like the respiratory pollutant asbestos fibers [26] or filamentous bacteria-escaping immune cells [27]. Fabrication methods include film-stretching, where spherical particles are embedded into films, liquefied with heat or plasticizer and then stretched into a variety of shapes and aspect ratios [28,29]. An example of a continuous method is particle replication in non-wetting templates (PRINT), where fluorinated molds shape the material into the designated form, followed by photochemical crosslinking [30]. This technique offers precise control over particle size and shape [31]. Despite many advantages, they are not suitable for the production of nano-in-micro particles [32], which are targeted in this study.

Aspherical, rod-like particles, due to their possibility to align with the airflow in pulmonary applications, are predicted to exhibit superior aerodynamic properties, which enables a deeper lung penetration compared to spherical particles [33,34]. This has been successfully observed in dry powder formulations with bigger and less uniform particles than in this study [35,36], as well as for nebulizer data [37], directly compared to non-elongated carriers. 

Altering the shape also affects the interaction with surrounding cells. Particles with high aspect ratios are taken up more slowly and in fewer amounts [38,39], resulting in reduced clearance for an extended release and absorption time. Additionally, for nano-in-micro carriers, non-cleared rods start to break down, allowing the resulting, free nano-particles to engage with immunologically active epithelial cells [40].

These advantages indicate that an aspherical, microparticulate drug delivery system composed of nanoparticles offers an innovative and promising approach for pulmonary applications, overcoming old flaws while merging the best features of different concepts. Therefore, this study aimed to fabricate rod-like, nano-structured microparticles of varying dimensions using a template-assisted approach [41,42] and capillary forces, aiming for efficient particle loading and stabilization. These micro-cylinders were morphologically characterized using an in situ imaging analysis tool (FlowCam^®^) and electron scanning microscopy (SEM). Silica nanoparticles were used as a model nano-particle system, and alginate was chosen as the stabilizer due to its reversible crosslinking, biocompatibility and minimal immunogenic responses [43]. This choice decreases the potential harmful effects of the carrier system, supported by its reversal gelation in body fluids [44], preventing toxic accumulation in the lungs. Different gel strengths and stabilizing steps were assessed on their stability to modulate the carrier’s disintegration speed. The aerodynamic properties of these particles were measured using an NGI and were compared to similar spherical particles. Rods of varying aspect ratios (ARs) were evaluated for toxicity on dTHP-1 cells using an MTT assay. Their potential to alter macrophage uptake and clearance was assessed through Confocal Laser Scanning Microscopy (CLSM). 

## 2. Materials and Methods

### 2.1. Materials

For the particle preparation, ipPore^TM^ track-etched polycarbonate membranes with a pore density of 1 × 10^6^/cm^2^, varying thicknesses (7, 12 and 22 μm) and a constant pore size of 3 μm were purchased from 4it4ip S.A. (Louvain-la-Neuve, Belgium). Spherical silica particles with a plain surface and fluorescent-green label (exc./em.: 485/510 nm) in 0.2, 3, 5 and 10 μm size were obtained from Kisker Biotech (Steinfurt, Germany). Sodium alginate, agarose, calcium chloride, and L-leucine were purchased from Sigma Aldrich (Steinheim, Germany). Tetrahydrofuran (THF) was obtained from Thermo Fisher Scientific Inc. (Darmstadt, Germany).

### 2.2. Cell Culture

Phosphate-buffered saline solution (PBS), Hanks’ Balanced Salt solution (HBSS), dimethyl sulfoxide (DMSO), Methanol, phorbol 12-myristate 13-acetate (PMA), 3-(4,5-dimethylthiazol-2-yl)-2,5-diphenyltetrazolium bromide (MTT), TritonX, and 4′,6-diamidino-2-phenylindol solution (DAPI) were obtained from Sigma Aldrich Life Science GmbH (Seelze, Germany) and Alexa Fluor^®^ 633 phalloidin was from Thermo Fisher Scientific Inc. (Darmstadt, Germany). Ibi-Treat^®^ microscopy chambers for CLSM imaging were acquired from Ibidi^®^ GmbH (Martinsried, Germany), and Cellstar^®^ 96-well plates were from Greiner Bio-One GmbH (Frickenhausen, Germany). The cells were cultured in RPMI-1640 medium supplemented with 10% fetal bovine serum (FCS) (Thermo Fisher Scientific Inc., Darmstadt, Germany) at stable conditions of 37 °C and 5% CO_2_. To differentiate the THP-1 cells into M0 macrophage-like cells (dTHP-1) for the experiments, 50 ng/mL PMA was added to the growth medium, and the cells were incubated for 48 h, washed with PBS and left to rest in fresh medium for 24 more hours [45]. As a quality control, the cells were microscopically examined, where an increase in cytoplasmic volume, enhanced adherence, and a flat, elongated, amoeboid morphology validated a successful differentiation [46]. 

### 2.3. Particle Preparation

The aspherical, cylindrical microparticles were produced using a template-assisted approach [41,42]. This method makes use of the defined shape and dimensions of membrane pores. These pores with a variable length of 7–22 µm and a width of 3 µm were infiltrated with fluorescent silica nanoparticles (SNPs) with a diameter of 200 nm, using capillary forces to achieve an effective and efficient infiltration. Briefly, 150 μL of a 1 mg/mL SNP suspension in distilled water was pipetted into a Petri dish and the membrane was placed on top, allowing the nanoparticles to be drawn up into the pores. Excess liquid was dried off under slightly increased temperature and airflow using a heater, and afterward, any residues were cleaned off with precision wipes. To ensure a complete filling, the process was performed 2 times from changing sides.

In a second step, hydrogels were used to interconnect the tightly stacked SNPs within the pores [47]. For an agarose crosslinking, a 1.5% agarose solution was heated up to 140 °C and filled into the template analogously to the nanoparticle loading. Using alginate, the membranes were first soaked in an alginate solution and then in a gelation liquid (100 mM CaCl_2_). The leftover gel was dried at 40 °C and cleaned off. 

The microrods were then released by dissolving the polycarbonate membrane in tetrahydrofuran and thoroughly washed. For NGI experiments, the resulting preparation was surface-coated with L-leucine to reduce the hygroscopicity and surface cohesiveness, leading to a more flowable powder [48,49]. For coating, the microrods were redispersed in an aqueous L-leucine solution and then freeze-dried. The amount of L-leucine was 0.4% of the mass of the rods. When necessary, the procedure was performed under aseptic conditions, ensuring an uncontaminated product for cell culture experiments.

### 2.4. Particle Characterization

To characterize the manufactured formulations and the spherical microparticles concerning their morphology, SEM was used. For this analysis, a Zeiss Evo HD 15 Electron Microscope (Carl Zeiss AG, Jena, Germany) equipped with a Lanthanum hexaboride (LaB6) cathode was applied. The sample to be analyzed was placed as a powder on a silica wafer and then coated with a gold layer about 10 nm thick to render the surface conductive, avoiding the accumulation of electrons. To generate the thin gold layer, a Quorum Q150R ES sputter coater (Quorum Technologies Ltd., East Grinstead, UK) was used. The micrographs were taken at a voltage of 5 kV and varying magnifications.

Particle counts (for the linearity of the method, see Figure S1) and geometric dimensions were obtained using a flow imaging microscope, which is proven to accurately depict aspherical particles within the desired size range [50]. The model utilized was the FlowCam^®^ 8000 series (Fluid Imaging Technologies, Scarborough, ME, USA), equipped with a FOV80 (80 μm depth and 700 μm width) flow cell, a 10× magnification objective and a 1.0 mL pump. The flow cell was rinsed with 1 mL cleaning solution (Citrajet^®^ cleanser, Fluid Imaging Technologies, Scarborough, ME, USA) and deionized water before each run. Measurement parameters were set to a flow rate of 0.1 mL/min and a camera rate of 19 frames/s for a sample volume of 100 μL, using the system’s auto-imaging mode with an auto-calibrated background. VisualSpreadsheet software version 4.18.5 (Fluid Imaging Technologies, Scarborough, ME, USA) was used to control the system and for data processing.

### 2.5. Disintegration Tests

To mimic the physiological conditions and the interstitial fluid in the upper airways, simulated lung fluid 1 (SLF1) was prepared as a release medium [51]. Next, 12 µm alginate–SNP–rods, produced with different concentrations of alginate gel (0.25, 1 and 4%), and a varying number of stabilization steps (1, 2 and 4 infiltration steps), were suspended and further diluted to a concentration of approximately 40,000 rods/mL. Separate micro-reaction tubes with 1 mL of suspension were incubated at 37 °C with slight shaking for various periods (0, 0.25, 0.5, 1, 2, 4, 8, 24 and 168 h). After each interval, the sample was analyzed with the FlowCam^®^, as previously described, to assess the number of disintegrated particles. Intact microcylinders were defined by parameters such as length, width, roughness and intensity (refer to Table S1 for the complete list) based on a library composed of ideal rods of non-disintegrated batches. Using the software’s filter method and a manual review, each sample’s amount of still-intact rods within a test series was compared to the corresponding timepoint 0 value, therefore concluding the percentage of disintegration.

### 2.6. Aerodynamic Particle Size Analysis

To estimate the deposition behavior of the inhalable preparation, the in vitro deposition and aerodynamic properties were determined, using a Next Generation Impactor (NGI) (Copley Scientific, Nottingham, UK). The experiment was performed with a setup described by Marple et al. [52] at a flow rate of 60 L/min, which was adjusted by a M1A flowmeter (Copley Scientific, Nottingham, UK) to ensure efficient aerosol formation of the different formulations [53,54]. A size-3 hard gelatin capsule was filled with 3 mg of the freeze-dried powder, placed inside a HandiHaler^®^ (Boehringer Ingelheim, Ingelheim, Germany) and punctured. Aerosolization of the particles was achieved by a 4 s gas flow produced by a vacuum pump (Erweka, Langen, Germany). Impacted powder in all NGI parts, the HandiHaler^®^, and the capsule were collected by rinsing with defined amounts of deionized water. The microparticle concentration of all samples was derived by measuring the fluorescence with a microplate spectrophotometer (TecanReader^®^ infinite M200, Tecan, Männedorf, Switzerland) at ex./em.: 485/510 nm (for the linearity of the method, see Figure S2). The results obtained were then used to calculate the Mass Median Aerodynamic Diameter (MMAD), the Fine Particle Fraction (FPF) and the Geometric Standard Deviation (GSD) according to Abdelrahim et al. [55] and as carried out before by our group [20].

### 2.7. Cell Viability

Cell viability was measured using a 3-(4,5-Dimethylthiazol-2-yl)-2,5-diphenyltetrazoliumbromid (MTT)-assay [56,57]. Cells of 5.0 × 10^4^ THP-1 were seeded per well of a 96-well plate in 200 μL medium and were cultivated and differentiated as previously described in Section 2.2. Subsequently, after washing with HBSS, positive (2% TritonX) and negative (RPMI-1640 + 10% FCS) controls, as well as different concentrations of the aspherical microparticles, were added. Concentrations of 0.005, 0.01, 0.05, 0.1 and 0.5 mg/mL, diluted in medium, were used and incubated for 4 and 24 h under careful shaking. After washing the cells with HBSS, 100 μL HBSS with 10% MTT reagent (5 mg/mL) was added and incubated for another 4 h. In the end, the supernatant was removed, 100 μL DMSO was used to dissolve the formazan crystals for 20 min, and the absorbance was measured at 550 nm by the TecanReader^®^. 

### 2.8. Cellular Uptake

To determine cellular uptake of the different microparticle species in macrophages, 4.0 × 10^4^ THP-1 cells in 300 μL medium were seeded per well on a µ-Slide 8 Well and differentiated as stated before. After washing twice with HBSS, 120,000 agarose-SNP rods of 7, 12 and 22 μm length as well as 3, 5 and 10 μm sized spherical microparticles were incubated for 24 h. Afterward, the cells were fixated with ice-cold methanol for 30 min, and the nuclei were stained with 300 nM DAPI for 20 min and F-actin with 5 µg/mL Alexa Fluor^®^ 633 phalloidin for 30 min with respective washing steps and finally suspended in PBS. Confocal laser scanning microscopy (CLSM) (LSM710, Carl Zeiss AG, Jena, Germany), equipped with 20×, 40× and 60× magnification objectives, was used for visualization, and ZEN blue 3.8 software (Carl Zeiss AG, Jena, Germany) was used for image processing. Lasers operating at wavelengths of λ = 488, 405 and 633 nm were employed to detect the rhodamine-green labeled silica particles, cell nuclei and cell membranes, respectively. For quantification of the cellular uptake, a particle-counting approach was chosen [58]. Samples of 250 ± 20 randomly selected cells were examined for internalization. To ensure that only genuinely internalized particles were counted, colocalization of the green fluorescent silica particles and red-stained F-actin was sought [22] (see Figure S3). Multiple planes were analyzed to validate this, incorporating periodic z-stacks as a quality control [59]. Additionally, as a negative control, cells were incubated at 4 °C as a metabolic uptake inhibitor [60]. 

### 2.9. Statistical Evaluation

Statistical analysis was performed with GraphPadPrism 10.0.1 (GraphPad Software, Boston, MA, USA). A sigmoidal 4PL curve fitting was applied for the disintegration experiments. To prove significance, ordinary one-way ANOVA was used for non-grouped data sets, and two-way ANOVA was used for grouped data, followed by Tukey’s HSD for multiple comparisons. *p* < 0.05 was considered significant (0.05 = *, 0.01 = **, 0.001 = ***, 0.0001 = ****). Data shown represent mean ± SD, *n* = 3 and N = 3 for biological experiments. 

## 3. Results and Discussion

### 3.1. Particle Characterization

**Figure 1:**
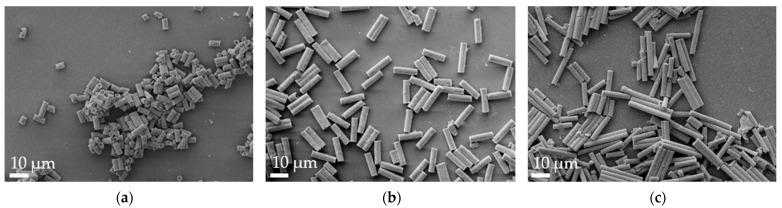
Representative alginate microrod batches, as visualized by scanning electron microscopy (SEM) at 2k× magnification. (**a**) 7 µm, (**b**) 12 µm and (**c**) 22 µm.

**Figure 2:**
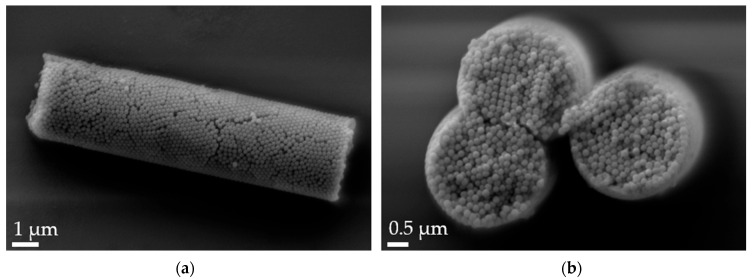
Scanning electron microscopy (SEM) micrographs of 12 µm alginate-stabilized microparticles. (**a**) Side view at 20k× magnification and (**b**) cross-section of a multi-rod assembly showing the arrangement of the nanoparticles and the thin alginate layer on top of the rods at 30k× magnification.

Aspherical microparticles, composed of spherical SNPs, were produced using template membranes of three different thicknesses (7, 12 and 22 μm). Capillary forces were utilized for rapid and efficient filling. The thickness, corresponding to the pore length, determines the resulting particle length, while a constant pore diameter of 3 μm ensures a uniform rod diameter. The structure was stabilized using the hydrogels alginate and agarose, respectively. As shown in the SEM images, nano-structured rods of different sizes were successfully fabricated, each with a consistent morphology and size within its respective group (Figure 1). The highly ordered submicron structure, including the stabilizing hydrogel bridges connecting individual nanoparticles, is evident at higher magnifications (Figure 2).

The flow-imaging analysis of the particles allowed us to measure a minimum of 5 × 10^3^ particles per sample (Table 1). This led to an average length determined of 7.67 ± 1.01 μm (R-7), 12.58 ± 1.88 μm (R-12) and 22.13 ± 3.40 μm (R-22) for alginate-stabilized particles. The results indicate that all particle fractions align closely with their template size. The mean diameter varied between 3.43 to 3.70 μm, which can be explained by the nature of the membranes: The process of creating pores via track-etching distributes them unevenly, meaning two or more pores can intersect each other. This leads to bigger structures with deviating geometric features, like a higher diameter. This can be reduced by choosing templates with lower pore densities, which then lowers the chance of multi-rods. Besides this membrane-specific issue, the swelling of the alginate gel might also lead to larger sizes in aqueous media. 

**Figure 3:**
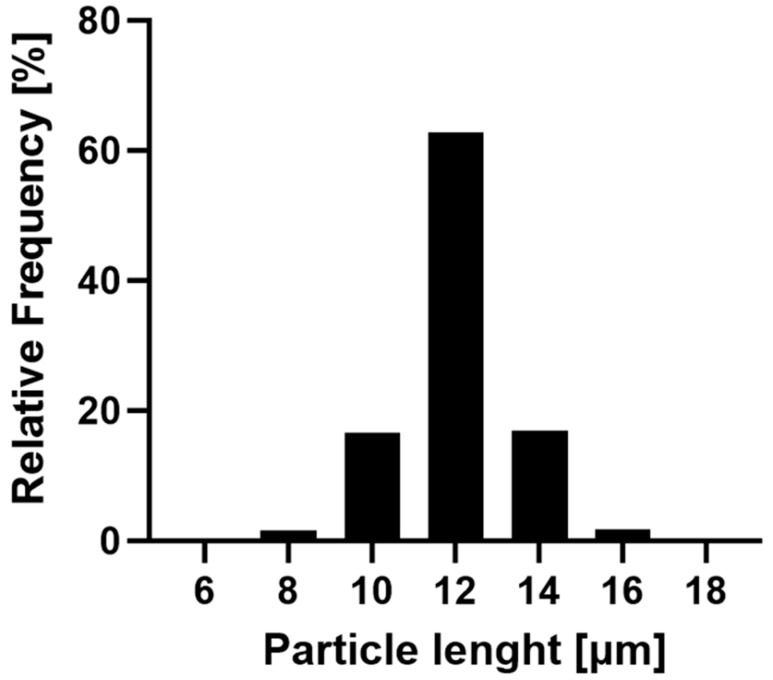
Frequency of the size distribution of the R-12 formulation. In total, 17,806 rods of three batches were evaluated with the FlowCam^®^, indicating a narrow distribution of particle sizes.

The R-12 alginate rods were exemplarily selected to showcase the narrow size distribution achieved by this fabrication method (Figure 3). The majority (96.48%) of the formulation falls within a 10 and 14 μm length, with over 60% of the particles aligning with the theoretical pore size. Only a minor fraction is smaller than 10 μm, which can be attributed to inadequately stabilized particles or compromised structures during the purification process. Larger particles are explained by the inherent characteristics of the membrane, where inclining or overlapping pores might result in dimensions exceeding the template’s nominal thickness. Additionally, nanoparticles can accumulate atop the pores while being stabilized, thereby surpassing the theoretical length. In conclusion, this production method yields nano-structured, aspherical microparticles of varying lengths in uniform size distribution and morphology.

### 3.2. Disintegration Tests

The disintegration behavior of the drug carrier was examined due to its important role in ensuring the release of the API which could be in the nanoparticles or loaded on the surface. Furthermore, it is also crucial to prevent long-term accumulation in the lungs, which could lead to increased toxicity or the activation of the inflammasome of the intact drug delivery system [61]. The ability to adjust the drug delivery systems’ breakdown is essential as well, whether it is to maintain the unique particle shape for extended periods with its special cell interactions to tune the uptake kinetics or to achieve a more rapid drug release.

The stability was assessed in SLF1 at 37 °C under slight shaking over the course of one week. The count of ideal particles regarding their morphology and various other characteristics was evaluated at each measurement interval and compared to the baseline at t = 0 h. This analysis primarily indicated the onset of each particle’s microstructural fragmentation rather than its total disintegration or dissolution. The flow-imaging method, utilizing the FlowCam^®^, can efficiently and reliably measure qualitative and quantitative attributes of aspherical particles [62], as well as track their respective changes (Figure S4). However, its capability is restricted to the lower micrometer range [50]. Thus, the described parameters serve as an indicator of the overall stability of the formulation.

Alginate as a stabilizer was chosen because of its biocompatible properties and low immunogenic responses [43]. Different formulations were used to assess the influence of stabilizing infiltration steps (1×, 2×, 4×) and gel concentrations (1%, 0.25%, 4%), where more steps and higher concentrations are assumed to provide better integrity to the structure. 

**Figure 4:**
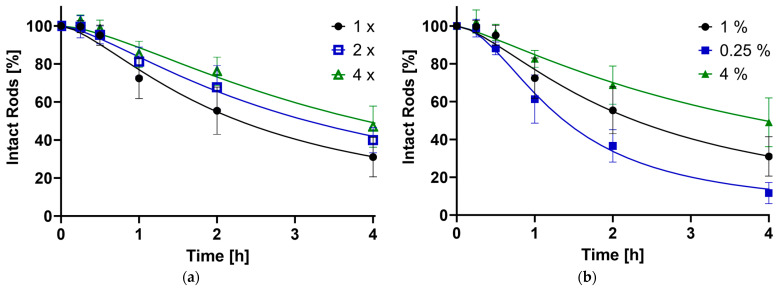
The percentage of still-intact rods compared to timepoint zero plotted against time in hours, as determined by the FlowCam^®^. (**a**) Influence of the number of stabilizing steps (1× = Standard, 2×, 4×); (**b**) Effect of the alginate concentration (1% = Standard, 0.25%, 4%).

As shown in Figure 4, the disintegration of the microrod population follows a sigmoidal-like curve, which is typical for alginate-based products [63]. This is explained by the physicochemical properties of the polysaccharide alginate: Polyguluronate blocks form so-called chelated egg-box structures in the presence of divalent cations such as calcium ions, leading to the formation of a stable gel, while the polymannuronate sequences just enter ionic bonds with the Ca^2+^ [64]. Introducing the gel into a medium containing monovalent cations such as Na^+^ as well as phosphate, an ion exchange starts to take place. At first, the more loosely, ionically bound calcium ions get released and captured by the PO_4_^3^, while the gel-forming structure stays intact; thus, a higher stability of the system in the first hour is observed. In the next stage, the egg-box structures get disrupted through an ion exchange with monovalent cations. Swelling, and subsequently, degradation and erosion of the alginate occurs [65,66]. This destabilizes the rod structure, leading to a fast loss of intact particles. Since this process is also dependent on the number of calcium ions in the medium [67], the degradation slows down with increasing content getting released from the alginate. Drying the gel completely, as done in this work, leads to the formation of more stable multimer egg-box structures [68], explaining completely intact rods after as long as a week. Furthermore, the stabilization process will always add a certain variety in the amount of alginate that sustains each rod structure, possibly adding thicker and stronger layers around certain fractions.

The stability of the drug carrier was influenced by the concentration of the stabilizer and the number of stabilizing alginate infiltration steps employed. An increase in the quantity of reinforcing procedures and a higher alginate concentration both contributed to a slower reduction of intact particles. Conversely, a lower alginate amount accelerated the reduction. This trend is visually represented in Figure 4, which includes timepoints of up to 4 h most relevant for pulmonary application due to the lung’s clearance mechanisms. The full disintegration curve can be found in the supporting data (Figure S5). The data sets were further analyzed, pinpointing specific time points at which 90% (t_90_), 50% (t_50_) and 10% (t_10_) of the rods remained intact relative to the initial population (Table 2).

For the standard formulation (1% alginate gel, 1× crosslinked), t_90_ was achieved at 0.49 h. The composition with 0.25% alginate trailed slightly, reaching the point at 0.41 h, suggesting the process behind the initial lag time always needs a certain amount of time. By increasing the stabilizing parameters, t_90_ can be extended by up to 1.8 times. In total, 50% and 10% of the rods were still intact after 2.20 and 9.77 h, respectively, for the default preparation. Reducing the alginate concentration approximately halved these durations, while enhancing the stabilization extended the times by factors of 1.4 to 1.8 for t_50_ and 1.4 to 2.2 for t_90_. The impact of stabilization steps appears to be smaller than the concentration. This can be attributed to additional handling and cleaning steps compromising the microstructure. Overall, the findings align with the initial hypothesis: a greater amount of stabilizer results in a more resilient structure over time.

The system is shown to be biodegradable, preventing harmful accumulation in the lungs. It can be adjusted to a faster disintegration of the carrier, facilitating a quicker release of its load, while preserving microstructures for sustained drug delivery. It is also possible to keep the shape intact for longer periods of time, to take advantage of particle–cell interaction as described in Section 3.5.

### 3.3. Aerodynamic Particle Size Analysis

For pulmonary application, the aerodynamic properties of the particles are of high relevance and were evaluated using an NGI, serving as an in vitro surrogate to conclude potential in vivo deposition patterns [69]. While aerodynamic properties are influenced by factors like aerodynamic size and individual airway characteristics, particle shape also plays a pivotal role [33,34]. Ensuring optimal aerodynamic properties is essential for efficient delivery to the lungs, thereby minimizing side effects, increasing patient compliance, and improving the overall cost-effectiveness of the treatment [70].

**Figure 5:**
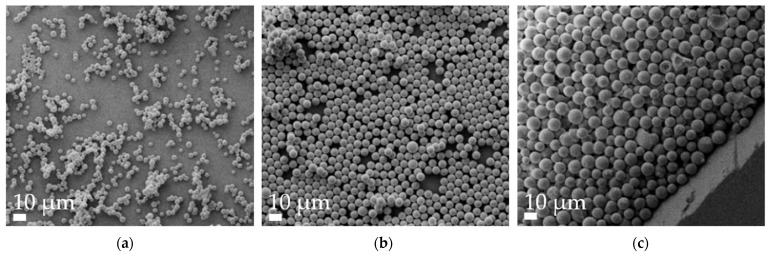
Representative SEM micrographs of spherical microparticle batches at 1k× magnification. (**a**) 2 µm, (**b**) 5 µm and (**c**) 10 µm.

Three different alginate-stabilized microrod formulations were tested and compared to spherical silica particles (Figure 5) similar in formulation-relevant parameters. The length of the delivery system varied between 7 (R-7, AR = 2.33), 12 (R-12, AR = 4.0) and 22 µm (R-22, AR = 7.33) whereas the diameter was kept constant at 3 µm. As for spherical particles, 3 µm (S-3) particles were chosen to represent an equal diameter, 5 µm (S-5), a similar volume, and 10 µm (S-10) resembling the length of the intermediate rod size. Slight changes (5 µm instead of 5.29 µm and 10 µm instead of 12 µm) were accepted because of commercial availability (for geometric dimensions of all formulations, see Table S2). 

**Figure 6:**
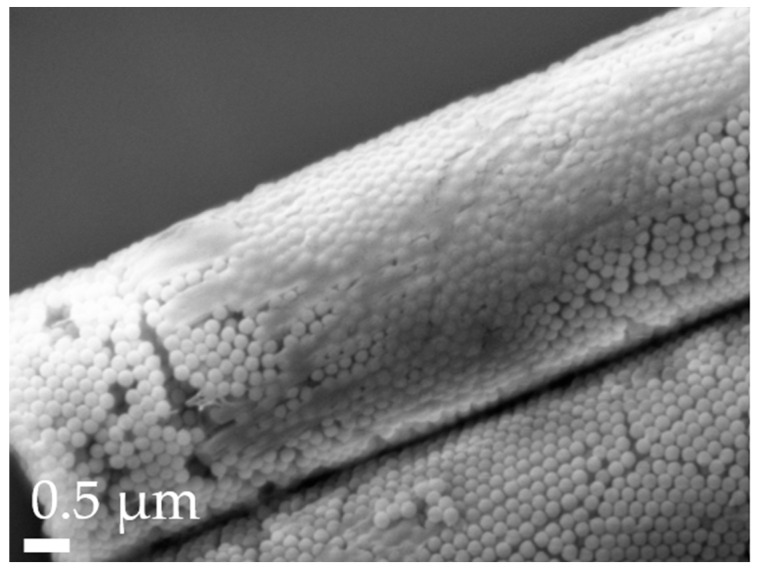
Close-up of L-leucine coating on an R-12 particle, as visualized by SEM with a 30k× magnification.

To improve the flowability of the powder formulations, and thus also modify the aerodynamic properties, the surfaces of all spherical and aspherical particles were coated with L-leucine. This proteinogenic amino acid forms a coating layer on the surface (Figure 6), reducing the interactions between the microparticles [71,72]. Thereby, agglomerate formation is reduced, which improves MMAD, GSD and FPF.

The results of the NGI analyses, as presented in Table 3, highlight that the aerodynamic properties of aspherical particles become less suitable for inhalation as their length increases. Specifically, the MMAD for R-12 stands at 3.62 ± 0.35 µm, with the smaller and bigger formulations deviating by roughly 30%. The FPF registers at 30.95 ± 2.25%, fluctuating by 3.91% and 7.64%, for the shorter and longer rods, respectively. Interestingly, when compared, the aerodynamic properties of spherical particles with similar relevant parameters are worse. The smallest particles (d = 3 µm) have an MMAD of 4.72 ± 0.18 µm, aligning with the 22 µm rods. This trend extends to the FPF, determined to be 24.19 ± 2.65%. This pattern persists, with the 10 µm spheres nearly doubling the MMAD. The fine particle fraction for all spherical particles significantly underperforms against the 12 µm aspherical particles as well. Across all formulations, the GSD slightly exceeds two.

The superior performance of the microrods compared to their spherical counterparts underscores the strong influence of particle shape. This is elucidated in the calculation of the aerodynamic diameter, which includes the so-called dynamic shape factor χ [73,74].

$$d_{a}=d_{ve}\sqrt{\frac{1}{\chi }\frac{\rho_{p}}{\rho_{0}}\frac{C_{c}(d_{ve})}{C_{C}(d_{a})}}$$

*d_a_*: Aerodynamic diameter

*d_ve_*: Volume-equivalent diameter

χ: Dynamic shape factor

*ρ_p_*: Particle density

*ρ_0_*: Standard density (1 g/cm^3^),

*C_c_*(*d_ve_*): Cunningham slip correction factor of the volume-equivalent diameter

*C_c_*(*d_a_*): Cunningham slip correction factor of the aerodynamic diameter

Given that χ is almost always larger than one for aspherical particles and equal to one for spherical particles [75,76], the value beneath the root diminishes for aspherical particles. This implies that for similar volume-equivalent diameters (*d_ve_*), the aerodynamic diameter is reduced. Consequently, the FPF, representing the fraction of mass that can enter the trachea–bronchial region with an aerodynamic size of <5 µm [77], is also directly dependent on this connection. To further elude this, each Cunningham slip correction factor was calculated (Equation (S2)). This factor corrects for a slip flow condition, where the interaction between gas molecules and the particle surface creates an irregular distribution of molecular impacts, leading to a deviation from Stokes’ law prediction of drag force, significant for particles <15 μm [78,79]. Afterward, the dynamic shape factors were computed based on the experimentally derived aerodynamic diameters (Table 4). These values are notably elevated compared to the assumed dynamic shape factor of spheres, with no significant differences among them. The observed trend of decreasing shape factor with increasing AR, which is inverse to the initial expectations, can be attributed to two primary factors: The larger surface area of the longer rods leads to higher agglomeration tendencies in air flow, as well as their higher susceptibility for early deposition due to interception, as discussed later in this section [3]. The spherical particles manifest a shift in the aerodynamic diameter towards larger values relative to their actual diameter, attributed to the density of the material (2.2 g/cm^3^) [80].In contrast, the nano-structure of the microrods leads to an MMAD reduction considering the maximal close-packing of equal spheres [81] and the polymer-filled space; therefore, a factor of 0.64 was applied to calculations regarding their density. 

This was further investigated with the calculation of the aerodynamic diameter (*d_a_*) using shape factors from the literature ([82], Table 4): The results revealed that while the differences between theoretical and experimental data for our experiments are not statistically significant, the rod formulations outperformed expectations. This can be attributed to the irregularity of the leucine coating (refer to Figure 6), which adheres more effectively to the rods due to their rough, textured, and larger surface and sticky hydrogel stabilizer. In contrast, the spherical particles do not benefit similarly from the coating and perform as anticipated. The superior performance of the R-7 and R-12 formulations compared to R-22 supports the previously postulated hypothesis about its technological challenges.

Because an MMAD below 5 µm is typically assumed to be necessary for deposition in the lower airways [83], it is concluded that all three rod formulations will reach the pertinent targets. Moreover, the efficacy of these particles is comparable to commercial DPI formulations using the HandiHaler^®^ [84,85] while being able to deliver higher amounts of API due to the larger volume of the delivery system.

Further analysis was carried out on the rods to understand their deposition pattern, based on their distribution across NGI stages. The results were grouped [86,87] and segmented as follows:

- Non-sized: big particles with extra-thoracic deposition (capsule, IP and pre-seperator).
- Coarse: pertaining to the upper respiratory tract, particle sizes between 4.46 and 12.80 µm (stages 1 and 2).
- Fine: associated with the bronchial region, covering particles between 0.94 and 4.46 µm (stages 3, 4 and 5).
- Extra Fine: relates to the alveoli, including particles smaller than 0.94 µm (stages 6, 7 and 8) [88].

**Figure 7:**
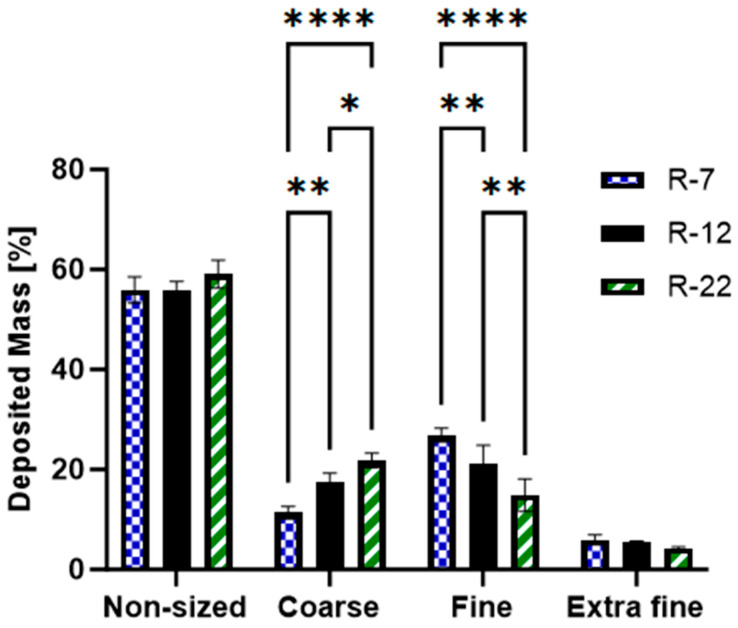
Grouped distribution pattern of the aspherical 7, 12 and 22 µm formulations, as measured by the NGI. *p* < 0.05 was considered significant (0.05 = *, 0.01 = **, 0.0001 = ****).

The data (Figure 7) revealed that both the 7 and 12 µm rods exhibited a ~55% non-sized deposition, with R-22 slightly higher at 59.1%. All formulations demonstrated less than 5% extra fine fraction with no significant variations. This pattern is to be attributed to the HandiHaler^®^’s design, which lacks an efficient dispersion mechanism [89]. The resulting necessary high flow rate, together with non-separated agglomerates, leads to a high non-sized deposition [90]. Particles below an MMAD of 0.94 µm are explained by minor fractures or free nanoparticles after the pressured airflow. Both fractions then elucidate the slightly increased GSD of around 2.2–2.4.

The most notable, significant differences were observed between the coarse and the fine category. The fraction of particles between 0.94 and 4.46 µm increased in the order of R-7 > R-12 > R-22, fluctuating between 26.8 and 14.9%. Exhibiting an inverse trend, the coarse segment ranged from 11.4 to 21.8%. The morphology of the microrods also contributes to this behavior, showing agglomerated and multi-rods in the non-sized fraction, and also indicates the respective deposition pattern, showing an increased object number in fine over coarse and especially extra fine (for exemplary SEM micrographs of rods after deposition for each group, see Figure S6).

Particles with a mean mass aerodynamic diameter exceeding 5 µm are postulated to deposit in the upper airways through inertial impaction. Those under 0.5 µm settle mainly in the deeper lung parts due to Brownian diffusion. Particles in between are predominantly affected by gravitational sedimentation [17,91]. For elongated particles like microcylinders or fibers, deposition by interception becomes a pivotal mechanism. In this process, the edges of these elongated particles come into direct contact with the lung surface, leading to their deposition [92,93]. The significance of this mechanism grows in relevance with increasing particle length. As the microrods become longer, they are more likely to brush the walls of the airways, especially in deeper lung departments, where the diameter diminishes. This phenomenon further explains the deposition pattern for rods of different lengths in various parts of the respiratory tract.

In conclusion, cylindrical microparticles, due to their unique shape, offer enhanced aerodynamic properties compared to similar spheres. While the performance tends to decline with increasing length, it remains within an acceptable range. This is particularly true when considering the increased delivered volume and surface, as a consequence of the augmented geometric dimensions per particle (Table S2).

### 3.4. Cell Viability

**Figure 8:**
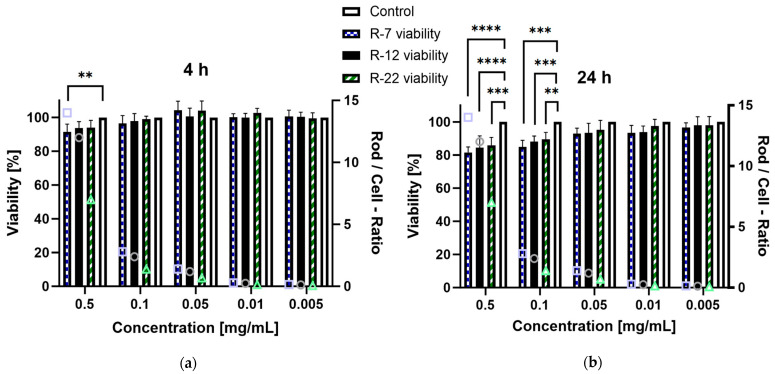
Viability of dTHP-1 cells in % after incubation with different concentrations of alginate-SNP rods of 7, 12 and 22 µm lengths. A secondary *y*-axis, depicting the number of rods per cell for each given concentration, was added for better comparison between the rod species (symbols: square = R-7, circle = R-12 and triangle = R-22). (**a**) 4 h and (**b**) 24 h incubation. *p* < 0.05 was considered significant (0.01 = **, 0.001 = ***, 0.0001 = ****).

The in vitro cytotoxic potential of the aspherical particles, stabilized with alginate, was assessed on dTHP-1 cells. This cell line serves as a model for human alveolar macrophages derived from monocytes [94], representing the primary defense mechanism of the deep lung regions as well as being a key target for drug delivery. Based on the NGI results from Section 3.3, a significant part of the inhaled formulations is going to interact with these immune cells; therefore, a toxicity evaluation is mandatory. Concentrations ranging from 0.001 to 0.5 mg/mL were evaluated over a 4 and 24 h incubation period. After 4 h exposure, no rod formulation but R-7 at 0.5 mg/mL exhibited a significant decrease in viability compared to the control, all exceeding 90% survival (Figure 8a). Extending the time to 24 h (Figure 8b), the trend persisted for concentrations up to 0.05 mg/mL. However, all rod lengths at 0.1 and 0.5 mg/mL displayed a significant decrease in viability compared to the control, though the lowest measured values remained above 80%, meaning no concentration led to a considerable decrease in cell viability [95].

Furthermore, the difference in cytotoxicity based on rod lengths was assessed, because the existing literature suggests an increased toxicity for particles with higher aspect ratios [96]. Due to the discrepancy in particle counts per milligram between differently sized rods, the number of rods in a sample divided by the number of cells was added as a ratio for easier visualization. The 0.1 mg/mL data point for the 22 µm rods was compared to the 0.05 mg/mL values for the 7 and 12 µm formulations as their closest match (a rod-to-cell ratio of circa 1.2–1.4). Interestingly no significant difference was observed for either timepoint, which is explained by differences in the uptake of the particles by dTHP-1 cells (see Section 3.5). In short, high aspect ratio particles are phagocytosed at a slower rate than their shorter counterparts, offsetting possible toxic effects. Overall, these findings indicate that the drug delivery system exhibits no relevant cytotoxicity towards the tested macrophages.

### 3.5. Cellular Uptake

The cellular uptake of both the aspherical and spherical microparticles of varying sizes was examined in dTHP-1 macrophages, following the methodologies as previously described by our group [22]. The main process for the internalization of large particles exceeding 0.5 μm is the actin-driven phagocytosis [97]. Alveolar macrophages, a subset of phagocytic immune cells, play a pivotal role in this context: They are the main clearance mechanism for objects that evade mucociliary clearance and resist dissolution in lung fluid or the lower airways [98]. Being an integral part of the lung’s immune response, they are also responsible for the production and release of cytokines [99]. Diseases like asthma or COPD can induce a continuous secretion of these proinflammatory mediators, inducing a lung-damaging chronic inflammatory state [5]. Given these implications, the interactions between the microparticles and macrophages are an important consideration. Because of shape-dependent differences in cellular uptake [38], various rod lengths (R-7, R-12 and R-22) were also compared to spheres with similar key properties in terms of diameter, surface area and volume (S-3, S-5, S-10), as described in Section 3.3.

**Figure 9:**
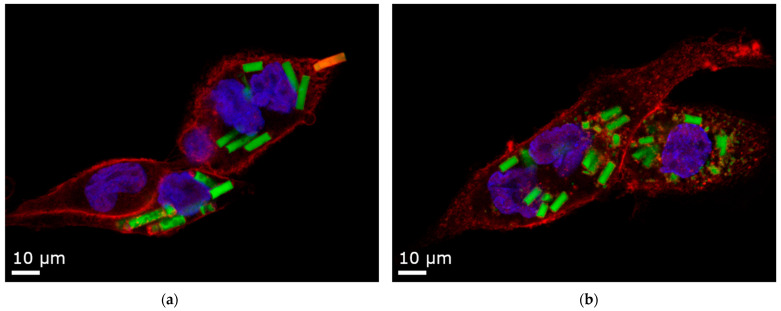
Confocal Laser Scanning Microscopy (CLSM) images of 12 µm rods uptaken by dTHP-1 cells. Red: F-actin staining with Alexa Fluor^®^ 633. Blue: nuclei staining with DAPI. Yellow: Rhodamine-green labeled silica particles. (**a**) Agarose stabilizer with intact microparticles and one rod in the process of being phagocytosed. (**b**) Alginate stabilizer, where the microparticles are partly disintegrated. For representative cell uptake images of all particle factions, refer to Figure S7.

A change was made regarding the stabilizing agent. Alginate microrods break down in ion-based media (as detailed in Section 3.2) or after macrophage uptake (Figure 9b), posing a challenge for the particle-counting approach. To address this and ensure intact morphology, agarose hydrogel was used as a substitute [100].

**Figure 10:**
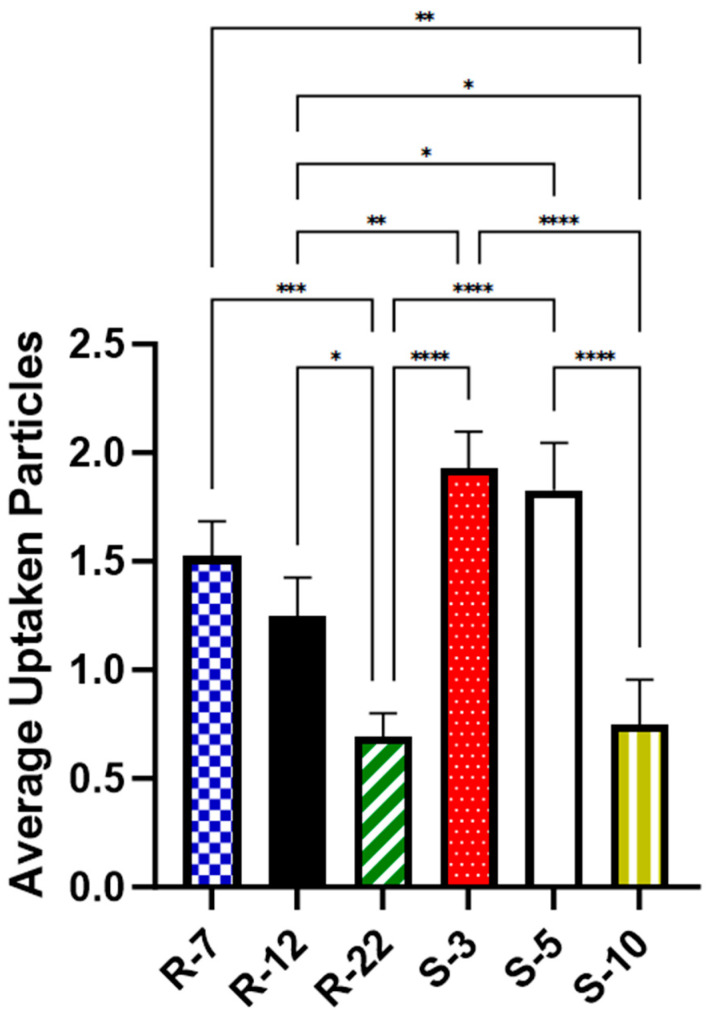
Average number of particles found inside dTHP-1 cells for various microparticle formulations, as quantified with CLSM after 24 h. *p* < 0.05 was considered significant (0.05 = *, 0.01 = **, 0.001 = ***, 0.0001 = ****).

As illustrated in Figure 10, approximately two spherical microparticles of sizes 3 μm (1.93 particles on average per cell, [p/c]) and 5 μm (1.83 p/c) were internalized on average per cell. In contrast, a significantly lower number of 10 μm spheres (0.75 p/c) were found inside macrophages, consistent with the existing literature [101,102]. For aspherical particles, slightly fewer 7 μm particles (1.52 p/c) were phagocytosed compared to spheres of a similar diameter. The uptake of 12 μm decreased significantly to about 0.7 times (1.25 p/c) when compared to their spherical counterparts in diameter and volume. The 22 micrometer rods (0.7 p/c) showed an even bigger decrease, significant with 0.56-fold compared to the medium-sized cylinders. The results highlight the expected important role of particle shape in cellular uptake, a trend that is more evident with increasing aspect ratios.

Phagocytosis can be conceptualized as a two-step process: initial particle attachment followed by an actin-driven envelopment, leading to its ingestion [103,104].

Initially, the attachment step considerably favors smaller particles [105]. This is facilitated by membrane ruffles, with the effect being even more pronounced for particles exceeding 6 μm in length, like elongated ones [106]. In the subsequent phase, the time taken for engulfment is greatly increased for aspherical particles. Even for particles significantly shorter than presented in this study, delays reach up to four–five times, extending the process up to an hour [26]. The efficiency of the uptake is highly dependent upon the curvature of the macrophage’s point of contact (Figure 9a: a rod is being phagocytosed, starting at its pointy side). Attachment at the flat side of a particle promotes the macrophage’s membrane to spread around it, extending the event, and not necessarily resulting in complete engulfment [107,108]. This phenomenon of frustrated phagocytosis is observed for longer particles [109] and is even more pronounced for a combination of a high AR with a substantial volume [110].

In summary, a significantly decelerated phagocytosis process and incomplete particle internalization explain the observed uptake hierarchy: spheres > short rods > big spheres ≈ long rods. This enables the drug carrier to be tailored specifically for its intended use: a routine uptake like regular, spherical particles, or it can be designed for escaping macrophage clearance, ensuring sustained drug delivery and a possible depot effect.

**Figure 11:**
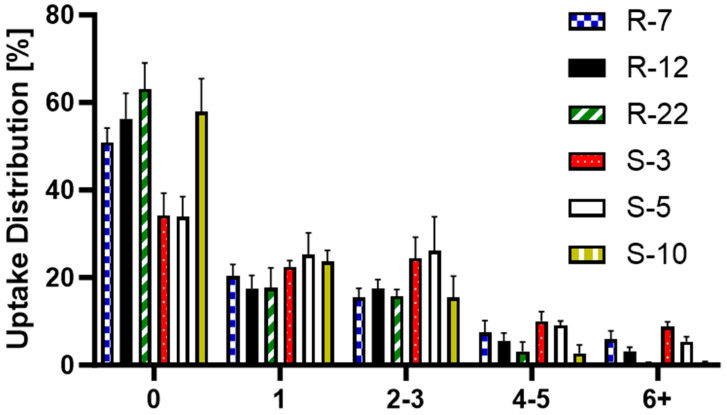
Post-uptake distribution of S-3, S-5, S-10, R-7, R-12 and R-22 in %.

Further analyzed was the particle distribution post-uptake (Figure 11) to evaluate the differences in cellular uptake between the various particle formulations, providing a deeper understanding of the mechanisms behind macrophage interactions with differently sized and shaped particles. S-3 and S-5 were found in a larger number of cells, with fewer than 40% of macrophages not having engulfed any. For all other formulations, 50–60% remained without particles, which can be explained by the heterogeneity of PMA-differentiated THP-1 cells [111]. The internalization of a single particle was consistent at around 20%. The trend changed slightly for two–three particles per cell, where S-3 and S-5 μm were found more frequently. For higher particle counts, the 10 μm spheres and 22 μm rods showed a noticeable drop, practically non-existent for six + particles in a single cell. R-12 also displayed notable differences for counts above three particles. Overall, individual macrophages internalized smaller spherical particles more frequently than aspherical ones. Among the microrods, uptake per cell decreased as length increased. They are mostly evenly distributed (one–three particles per cell), indicating a more uniform drug delivery to macrophages compared to spherical particles. Building on the previous conclusion, dTHP-1 cells with an average diameter of 15 μm are also limited by volume for large quantities of big particles [112].

**Figure 12:**
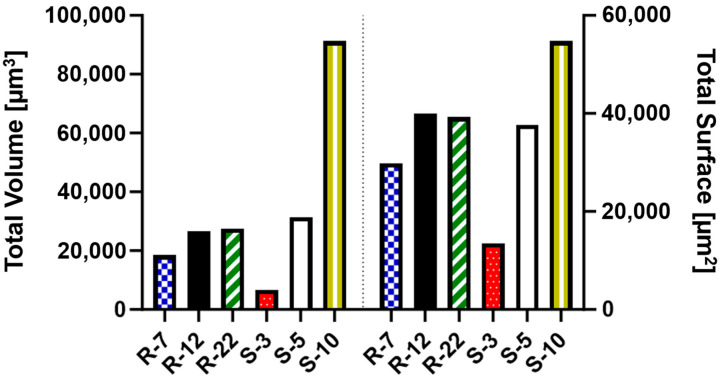
Total uptaken volume and surface as a measure for the potentially loaded drug amount for various microparticle formulations.

Additionally, the amount of internalized surface and volume of each microparticle formulation was analyzed. This comparison is key to understanding the impact of their geometric properties (Table S2) on effective API delivery to macrophages. As depicted in Figure 12, S-3 particles, despite high internalization numbers, account for a considerably smaller amount of volume and surface compared to the other formulations. The S-10 particles, while offering the highest potential for API delivery, demonstrate poor aerodynamic performance in NGI experiments (see Section 3.3), rendering them unsuitable for pulmonary delivery. In contrast, both S-5 and the microrods achieve a substantial uptake volume (20,000–30,000 μm^3^) and surface area (30,000–40,000 μm^2^). Notably, the presented cylindrical drug delivery system exhibits larger deposition in respective areas of the NGI due to their favorable aerodynamic properties. Their design allows for API loading on both the surface and within the nano-structure, enabling a high potential for API delivery. In conclusion, microrods are not only versatile and modifiable in their uptake behavior, but also excel in delivering substantial amounts of API to their potential targets.

## 4. Conclusions

The increased need for efficient pulmonary drug delivery has made the exploration of novel drug carriers necessary. By exploiting the particle shape and size in the micron range, combined with the advantages of nanoparticles and a high loading capacity, a strategy develops to overcome existing and emerging challenges.

Through a template-assisted approach, rods of varying lengths were successfully produced, exhibiting a tight size distribution and easily adjustable geometric features. The incorporation of biocompatible alginate gel as the stabilizer ensures the carrier’s tunable breakdown in body fluids. This flexibility provides either a rapid release, capitalizing on the benefits of its nanoparticulate load, or a prolonged depot effect. When compared to similar spherical particles, the aerodynamic properties of these rods showed significant improvements, leading to the potentially higher delivery of active pharmaceutical ingredients (APIs) to relevant lung areas. This is even more pronounced when considering the high volume and surface area of the drug carrier system. Notably, it demonstrated no significant toxicity toward THP-1 macrophages. The crucial role of particle shape in macrophage clearance was also highlighted, where an increased aspect ratio and volume resulted in fewer phagocytosed particles.

This study provides a more refined understanding of the presented drug carrier system, emphasizing its tunable geometric features and disintegration behavior. Further insights into the shape-dependency of aerodynamic properties and cellular interactions enable the leveraging of the systems’ highly adjustable features. This allows for the optimization of specific therapeutic needs while ensuring a high API delivery, advancing the field of innovative pulmonary drug delivery towards a more effective and individualized approach. To further progress this research, the rods need to be loaded with an API, for instance as a reactive oxygen scavenger being applied in inflammatory diseases, which might also influence the drug delivery system’s technological performance. Moreover, the reliance on in vitro models may not fully reflect complex in vivo environments, despite being insightful for this study’s intention. Additionally, the production process, while being effective in a small-scale laboratory setting, faces problems in sustainability due to the non-recyclable templates, though non-destructive methods are a research focus of this group.

## Figures and Tables

**Table 1:** Average length and width of various rod formulations as measured by the FlowCam^®^. At least 5000 particles per sample were analyzed.

Formulation	Length [µm]	Width [µm]
R-7	7.84 ± 0.59	3.46 ± 0.38
R-12	12.65 ± 1.16	3.43 ± 0.77
R-22	22.44 ± 0.62	3.70 ± 0.86

**Table 2:** Timepoints of different formulations at which 90%, 50% and 10% of rods are still intact compared to baseline point t = 0 h. t_50_ readings were obtained via the curve fit by GraphPad Prism 10.0.1 and t_90_ and t_10_ values were computed from the t_50_ and respective Hill slopes (Equation (S1)).

Formulation	t_90_ [h]	t_50_ [h]	t_10_ [h]
Standard (1%, 1×)	0.49	2.20	9.77
2×	0.65	3.04	14.07
4×	0.88	3.75	15.94
0.25%	0.41	1.33	4.29
4%	0.79	3.89	21.89

**Table 3:** Aerodynamic properties (MMAD, GSD and FPF) of aspherical (R) and spherical (S) microparticles of different sizes, calculated based on NGI experiments. Significance was tested against R-12. *p* < 0.05 was considered significant (0.001 = ***, 0.0001 = ****).

Formulation	MMAD [µm]	GSD	FPF [%]
R-7	2.88 ± 0.23	2.49 ± 0.16	34.27 ± 2.95
R-12	3.62 ± 0.35	2.27 ± 0.11	30.95 ± 2.25
R-22	4.72 ± 0.48	2.26 ± 0.13	23.31 ± 2.86 ****
S-3	4.72 ± 0.18	2.32 ± 0.17	24.19 ± 2.65 ***
S-5	7.11 ± 0.02	2.10 ± 0.03	16.92 ± 4.51 ****
S-10	10.95 ± 0.85 ****	2.66 ± 0.10	14.34 ± 1.90 ****

**Table 4:** The calculated and literature data for the shape factor χ. Furthermore, based on the literature values for χ, the aerodynamic diameter (*d_a_*) of the formulations was calculated.

Formulation	χ (Calculated)	χ (Literature, [82])	*d_a_* [µm]
R-7	3.49 ± 0.54	1.75	4.18
R-12	3.26 ± 0.60	2.25	4.47
R-22	2.78 ± 0.56	3.0	4.65
S-3	/	1	4.49
S-5	/	1	7.42
S-10	/	1	14.83

## Data Availability

Data are contained within the article and Supplementary Materials.

## Supplementary Materials

The following supporting information can be downloaded at: https://www.mdpi.com/article/10.3390/pharmaceutics16020232/s1, Figure S1: Linearity of quantitative rod measurements with the FlowCam^®^. y = 4,812,346 × x + 593.2, R^2^ = 0.9997 (mean y value of each point). Table S1: Glossary of particle properties and their definitions used with the FlowCam^®^, adapted from VisualSpreadsheet 5 User Guide Version A 2020. Figure S2: Linearity of the fluorescence measurements for the NGI experiments. y = 14,057 × x − 148.2, R^2^ = 0.9986 (mean y value of each point). Figure S3: Representative single channel and merge R-12 cell uptake images at the 24 h timepoint, as visualized by CLSM. The same gamma, black and white settings were used to highlight the differences between uptaken rods with a colocalization of the green and red fluorescence (a–d) and the negative 4 °C control (e–h). (a,e): DAPI core staining. (b,e): Alexa Fluor^®^ 633 actin staining. (c,g): Rhodamine-green labeled silica particles. (d,h): Merged images. Figure S4: Alginate rods of 12 µm captured by the FlowCam^®^. The number beneath each image represents the filter score, indicating the particle’s resemblance to an ideal microrod. (a) Intact particles and (b) after the start of disintegration. Figure S5: The percentage of still-intact rods compared to timepoint zero plotted against the time in hours (up to 168 h), as determined by the FlowCam^®^. (a) Influence of the number of stabilizing steps (1× = Standard, 2×, 4×); (b) Effect of the alginate concentration (1% = Standard, 0.25%, 4%). Equation (S1): Computing t_F_ values from t_50_ and Hill slope. Table S2: Geometric features (aspect ratio, surface and volume) of aspherical (R) and spherical (S) microparticles of different sizes. Equation (S2): Calculation of the Cunningham slip correction factor. Figure S6: Representative alginate microrod batches after NGI deposition, as visualized by scanning electron microscopy (SEM) at 2k× magnification. (a) Non-sized, (b) coarse, (c) fine and (d) extra fine. Figure S7: Representative cell uptake images after 24 h, as visualized by CLSM. (a) R-7, (b) R-12, (c) R-22, (d) S-3, (e) S-5 and (c) S-10.
