# Supplementary material for: Aspherical, Nano-Structured Drug Delivery System with Tunable Release and Clearance for Pulmonary Applications
Source: Pharmaceutics. 2024 Feb 5;16(2):232. doi: 10.3390/pharmaceutics16020232 (PMC10891959; doi:10.3390/pharmaceutics16020232)
Supplement: Supplementary file 1 [file pharmaceutics-16-00232-s001.zip › pharmaceutics-2777833-supplementary.pdf]

pharmaceutics

Article

Aspherical, nanostructured drug delivery system with tuneable release and clearance for pulmonary applications

Tomas Pioch [1], Thorben Fischer [1,2] and Marc Schneider [1,*]

[1] Department of Pharmacy, Biopharmaceutics and Pharmaceutical Technology, Saarland University, Saarbrücken, Germany; tomas.pioch@uni-saarland.de
[2] Current affiliation, Ursapharm Arzneimittel GmbH, Saarbrücken, Germany; thorben_fischer@gmx.net
* Correspondence: Marc.Schneider@uni-saarland.de; Tel.: +49 681 302 2438

1. Supplementary Materials

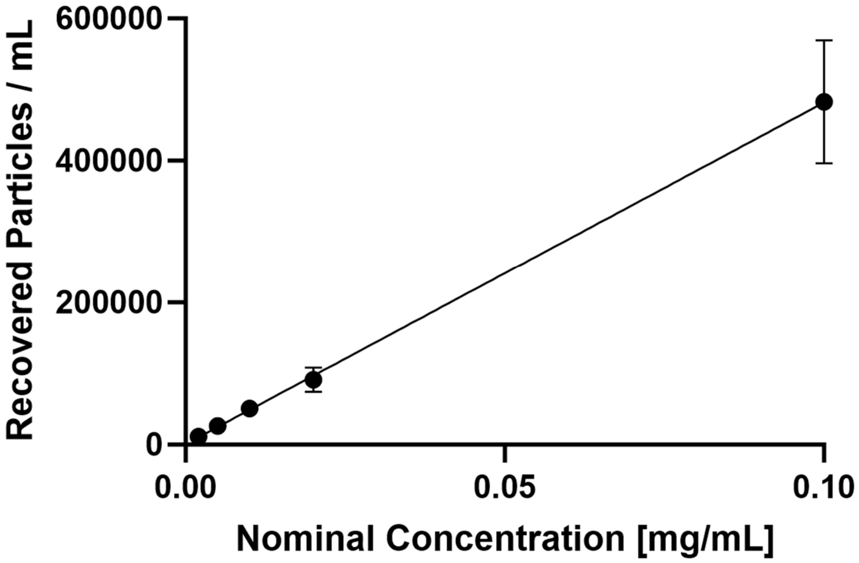

CC BY

Figure S1. Linearity of quantitative rod measurements with the FlowCam®. y = 4812346 * x + 593.2, R^2 = 0.9997 (mean y value of each point).

Table S1. Glossary of particle properties and their definitions used with the FlowCam®, adapted from VisualSpreadsheet 5 User Guide Version A 2020.

Particle property	Definition
Area	Number of pixels in the threshold.
Aspect Ratio	The ratio of the lengths of the axes of the Legendre ellipse of inertia of the particle.
Circle Fit	Deviation of the particle edge from a best-fit circle, normalized to the range [0,1] where a perfect fit has a value of 1.

Circularity (Hu)	"A Hu moment invariant as a shape circularity measure" by Zunic, Hirota, and Rosin, Pattern Recognition 43 (2010) pp 47-57)
Diameter (ABD)	The diameter based on a circle with an area that is equal to the ABD Area.
Diameter (ESD)	The Mean value of 36 feret measurements.
Edge Gradient	Average intensity of the pixels making up the outside border of a particle.
Feret Measurement	The perpendicular distance between parallel tangents touching opposite sides of the particle.
Holes	"Holes" may occur in binary particle images where particles have some degree of transparency, due to the center of the particle being "lighter" and closer to the background gray scale level.
Intensity	The average grayscale value of the pixels making up a particle.
Length	The maximum value of 36 feret measurements.
Roughness	A measure of the unevenness or irregularity of a particle's surface-the ratio of perimeter to convex perimeter.
Sigma Intensity	Standard deviation of grayscale values.
Sum Intensity	Sum of grayscale pixel values.
Symmetry	A measure of the symmetry of the particle about its center.
Width	The minimum value of 36 feret measurements.

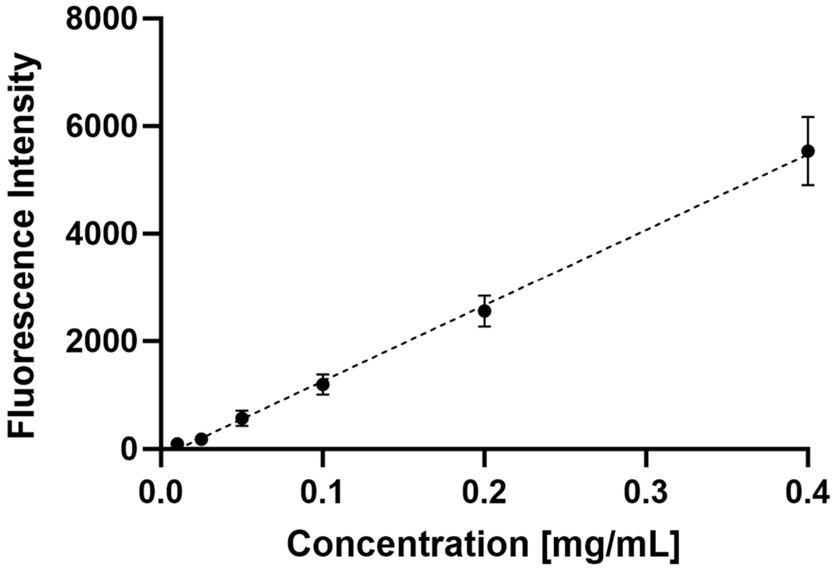

Figure S2. Linearity of the fluorescence measurements for the NGI experiments. y = 14057 * x – 148.2, R^2 = 0.9986 (mean y value of each point).

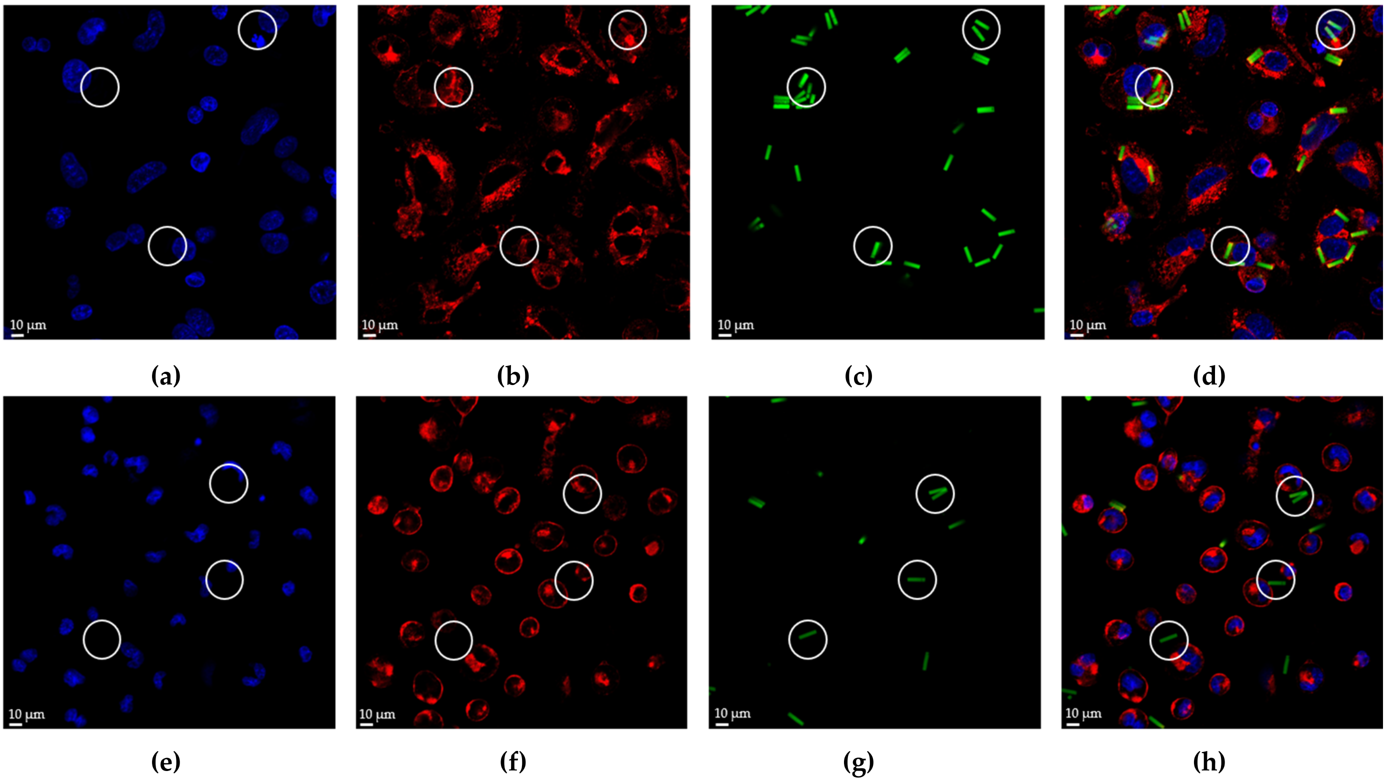

Figure S3. Representative single channels and merged image for R-12 cell uptake images at the 24 h timepoint, as visualized by CLSM. The same gamma, black and white settings were used to highlight the differences between uptaken rods with a colocalization of the green and red fluorescence (**a**–**d**) and the negative 4 °C control (**e**–**h**). (**a**) and (**e**): DAPI core staining. (**b**) and (**e**): Alexa Fluor® 633 actin staining. (**c**) and (**g**): Rhodamine-green labeled silica particles. (**d**) and (**h**): Merged image.

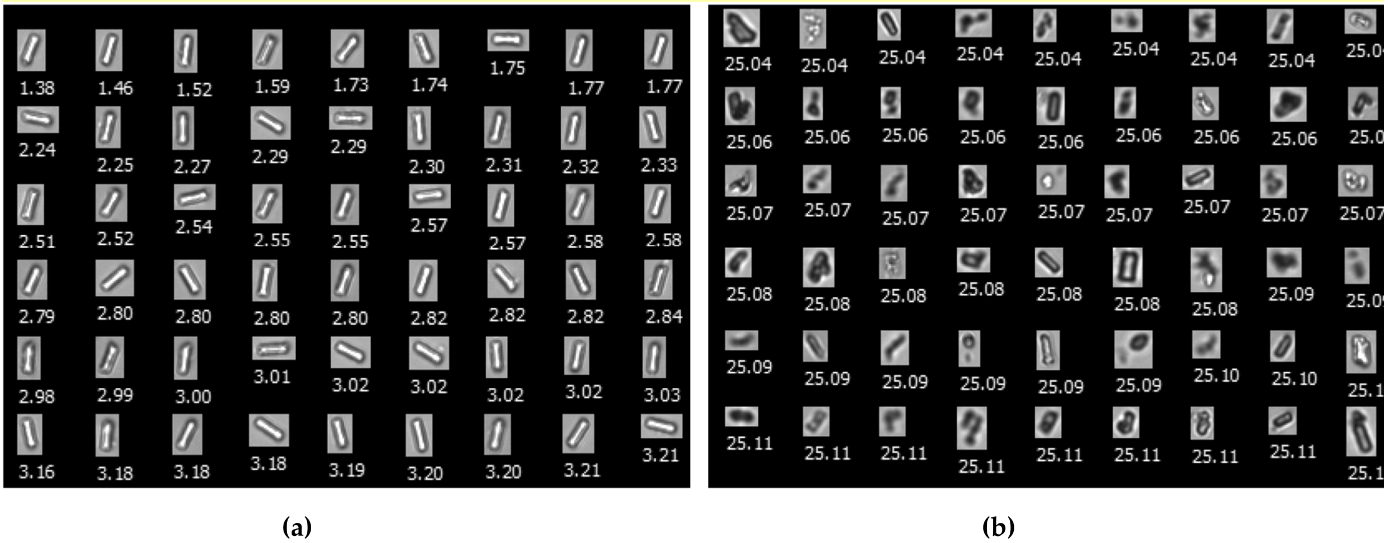

Figure S4. 12 µm alginate rods captured by the FlowCam®. The number beneath each image represents the filter score, indicating the particle's resemblance to an ideal microrod (filter score = 1.00). (**a**) Intact particles and (**b**) after the start of disintegration.

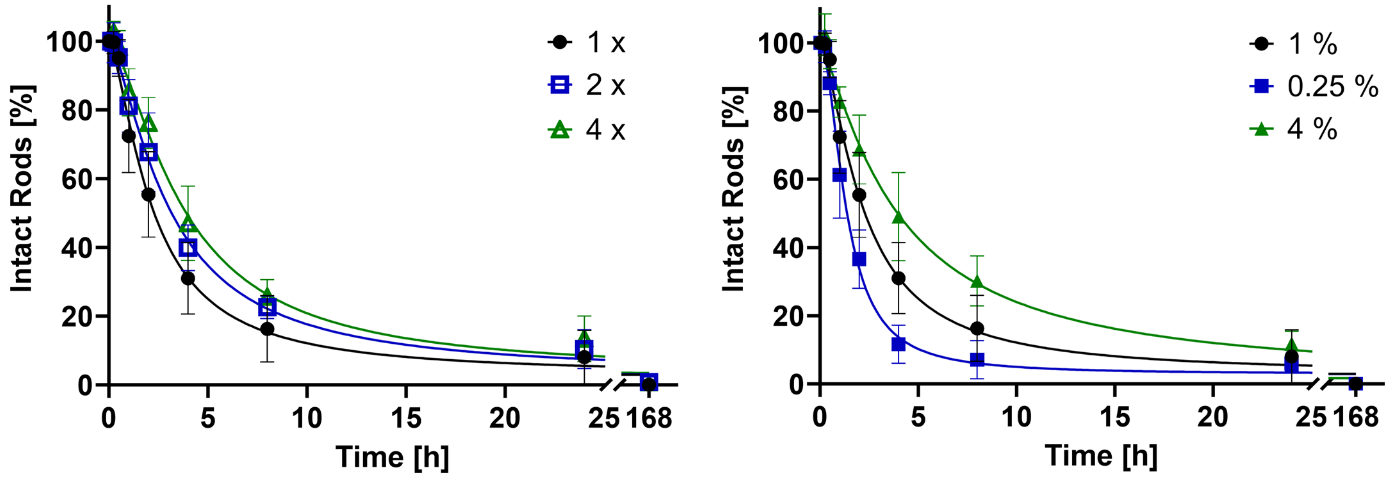

(**a**) (**b**)

Figure S5. The percentage of still intact rods compared to timepoint 0 plotted against the time in hours (up to 168 h), as determined by the FlowCam®. (**a**) Influence of the number of stabilizing steps (1x = Standard, 2x, 4x); (**b**) Effect of the alginate concentration (1% = Standard, 0.25%, 4%).

Equation S1: Computing t_F values from t_{50} and Hill slope.

$$t_F = \left(\frac{F}{100 - F}\right)^{\frac{1}{H}} \times t_{50}$$

t_F: Time point, where F rods are still intact
F: % intact rods
H: Hill slope

Table S2. Geometric features (aspect ratio, surface area and volume) of aspherical (R) and spherical (S) microparticles of different sizes.

Formulation	Aspect ratio	Surface area [µm²]	Volume [µm³]
R-7	2.33	80.11	49.48
R-12	3.67	127.24	84.82
R-22	7.33	282.34	241.28
S-3	1.00	28.27	14.14
S-5	1.00	78.54	65.45
S-10	1.00	314.16	523.59

Equation S2: Calculation of the Cunningham slip correction factor.

$$C_C = 1 + \frac{2\lambda}{d}\left(A_1 + A_2^{\frac{-A_3 d}{\lambda}}\right)$$

C_c: Cunningham slip correction factor
λ: Mean free path (0.066 µm at 20 °C [101])

d: Particle diameter
A_n: Experimentally determined coefficients (A_1 = 1.257, A_2 = 0.400, A_3 = 0.55 [102])

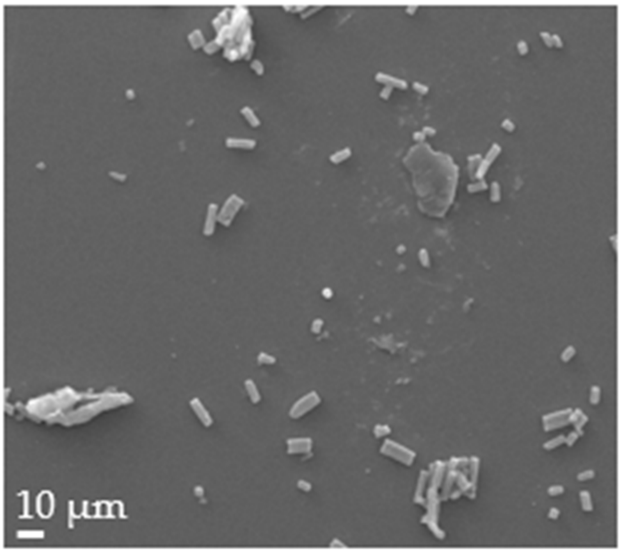

(a)

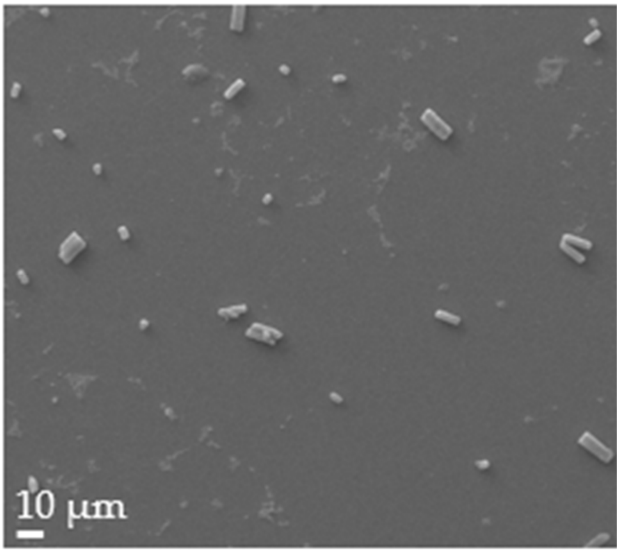

(b)

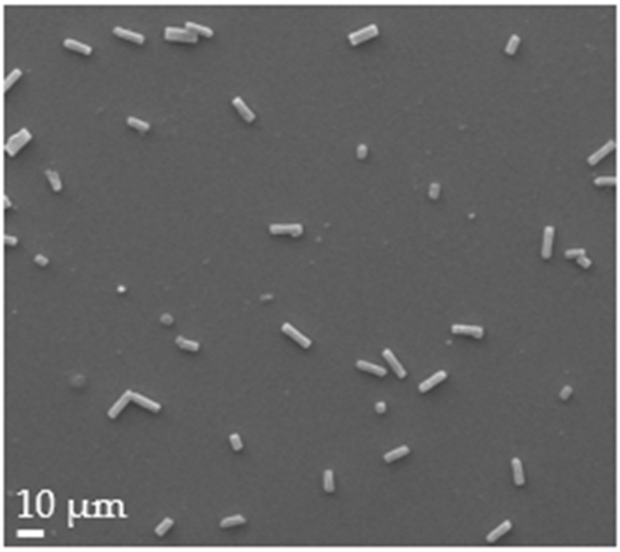

(c)

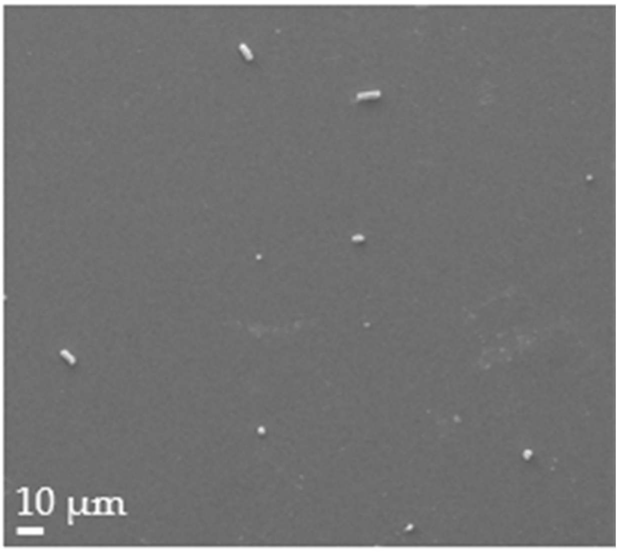

(d)

Figure S6. Representative alginate microrod batches after NGI deposition, as visualized by scanning electron microscopy (SEM) at 2 kX magnification. (**a**) Non-sized, (**b**) coarse, (**c**) fine and (**d**) extra fine.

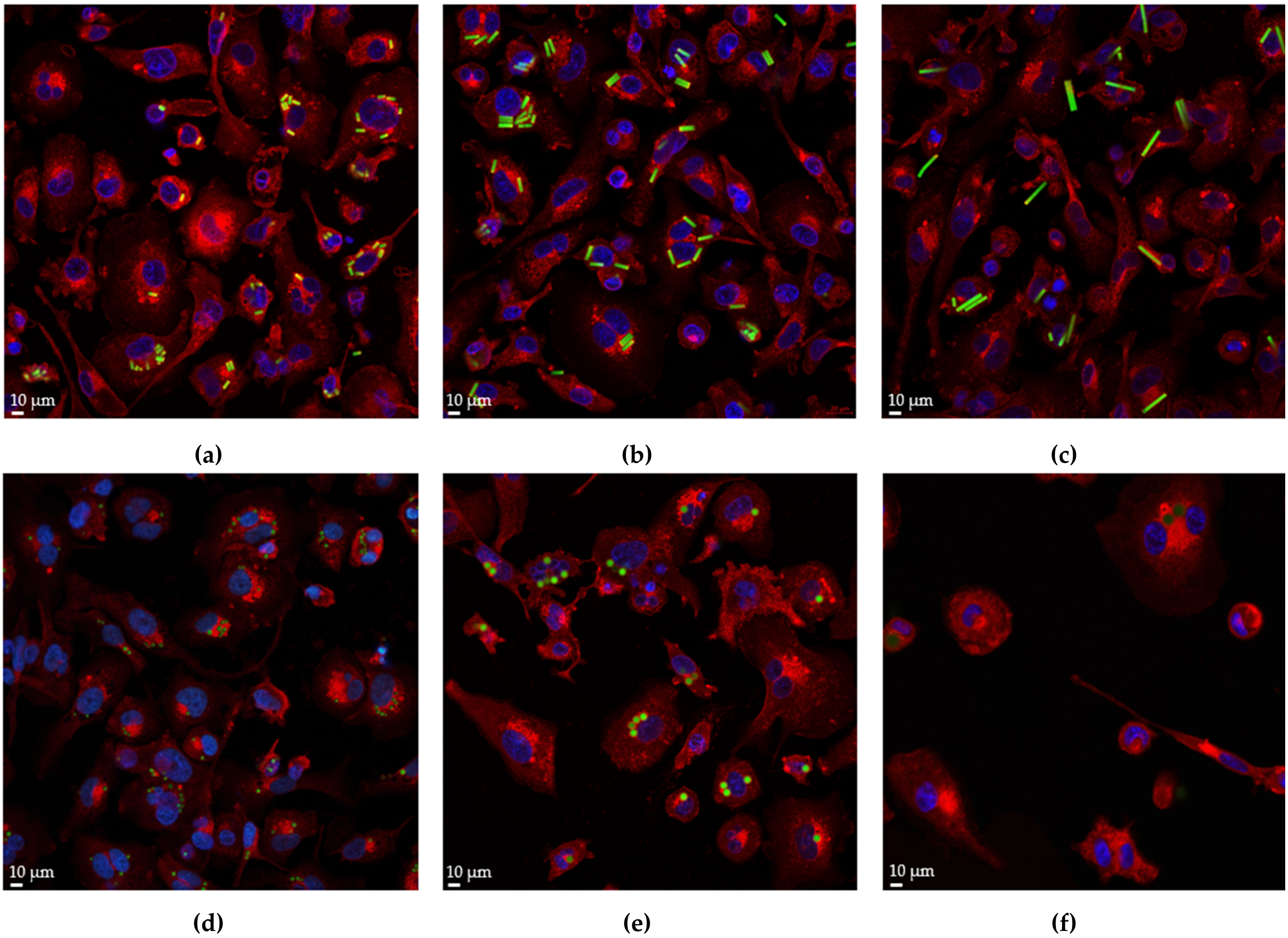

Figure S7. Representative cell uptake images after 24 h, as visualized by CLSM. (**a**) R-7, (**b**) R-12, (**c**) R-22, (**d**) S-3, (**e**) S-5 and (**c**) S-10.

Disclaimer/Publisher's Note: The statements, opinions and data contained in all publications are solely those of the individual author(s) and contributor(s) and not of MDPI and/or the editor(s). MDPI and/or the editor(s) disclaim responsibility for any injury to people or property resulting from any ideas, methods, instructions or products referred to in the content.
